# Notaufnahmebesuche von Pflegeheimbewohnern: Analyse von Routinedaten einer städtischen Klinik der Schwerpunktversorgung

**DOI:** 10.1007/s00063-022-00952-2

**Published:** 2022-09-07

**Authors:** Falk Hoffmann, Kirsten Habbinga

**Affiliations:** 1 Department für Versorgungsforschung, Carl von Ossietzky Universität Oldenburg, Ammerländer Heerstr. 140, 26129 Oldenburg, Deutschland; 2 Pius-Hospital, Medizinischer Campus der Carl von Ossietzky Universität Oldenburg, Oldenburg, Deutschland

**Keywords:** Pflegeheim, Notaufnahme, Krankenhaus, Triage, Tod, Residential facilities, Emergency department, Hospital use, Triage, End of life

## Abstract

**Hintergrund:**

In den letzten Jahren nimmt die Inanspruchnahme von Notaufnahmen in Deutschland zu, insbesondere durch ältere und pflegebedürftige Personen.

**Ziel der Arbeit:**

Es werden Notaufnahmebesuche von Pflegeheimbewohnern hinsichtlich ihrer Charakteristika und Symptome, der Diagnostik und des stationären Verlaufs untersucht.

**Material und Methoden:**

Es wurden Routinedaten eines Krankenhauses der Schwerpunktversorgung ohne Unfallchirurgie ausgewertet (Pius-Hospital Oldenburg). Aus dem Krankenhausinformationssystem wurden alle Notaufnahmebesuche von Pflegeheimbewohnern im Zeitraum 06/2014 bis 05/2017 selektiert.

**Ergebnisse:**

Von 47.270 Notaufnahmebesuchen entfielen 1676 (3,6 %) auf Pflegeheimbewohner (mittleres Alter: 78,8 Jahre; 62,9 % weiblich). Insgesamt 20,1 % der Notaufnahmebesuche waren an Wochenenden und 80,6 % erfolgten zwischen 8–20 Uhr. Durch Vertragsärzte wurden 40,3 % eingewiesen. Insgesamt 84,2 % der Bewohner wurden stationär aufgenommen, die Verweildauer war genau einen Tag bei 21,1 % und 2–3 Tage bei weiteren 14,6 %. Mit längerer Verweildauer stieg der Anteil der Einweisungen durch Vertragsärzte. Von allen stationär aufgenommenen Bewohnern verstarben 10,3 % während des Krankenhausaufenthalts (davon 28,2 % am Aufnahmetag und 47,9 % binnen der ersten 3 Tage). Verstorbene waren älter, die Notaufnahmebesuche waren häufiger am Wochenende und eine Einweisung durch Vertragsärzte erfolgte seltener.

**Diskussion:**

Die Studie zeigt Probleme auf verschiedenen Seiten. In Heimen sollten eine bessere interprofessionelle Kooperation mit Hausärzten sowie eine Stärkung der Palliativstrukturen erfolgen. In Notaufnahmen sollten Prozesse etabliert werden, die eine weitere ambulante Versorgung dieser Patientengruppe ermöglichen.

**Zusatzmaterial online:**

Zusätzliche Informationen sind in der Onlineversion dieses Artikels (10.1007/s00063-022-00952-2) enthalten.

## Abstract (en)

**Background:**

The number of emergency department (ED) visits in Germany continues to increase, especially by older people and persons receiving care.

**Objectives:**

We aimed to characterize ED visits by nursing home residents (NHR) in Germany.

**Materials and methods:**

We analyzed routine data of a hospital without trauma surgery (Pius-Hospital Oldenburg). All ED visits by NHR in the period 06/2014–05/2017 were selected from the hospital information system.

**Results:**

Of 47,270 ED visits, 1676 (3.6%) were NHR (mean age: 78.8 years; 62.9% women; 40.3% were referred). Of them, 20.1% were on weekends and 80.6% occurred between 8 am–8 pm. A total of 84.2% visits led to hospitalization; the length of stay was 1 day for 21.1% and 2–3 days for another 14.6%. With longer length of stay, the proportion of admissions by referring physicians increased. Of those admitted, 10.3% died during hospitalization (28.2% on the day of admission; 47.9% within the first 3 days). People who died were older, their visits were more frequently on weekends, and they were less often referred.

**Conclusion:**

Our study reveals problems in several areas. Improved interprofessional cooperation with general practitioners and strengthened palliative structures should be implemented in nursing homes. Furthermore, there is a need for establishing processes in EDs to realize continued outpatient care for these patients.

## Hintergrund

Durch den demographischen Wandel steigt auch die Anzahl älterer Patienten in deutschen Notaufnahmen [11]. Insbesondere die häufig vorhandene geriatrietypische Multimorbidität in Verbindung mit einem hohen Komplikationsrisiko, die schwierige Abgrenzung akuter von bereits vorliegenden Beschwerden sowie die Sicherstellung von therapierelevanten Informationen stellen bei diesen Patienten besondere Herausforderungen dar [21]. Dies gilt umso mehr bei Pflegeheimbewohnern. In Deutschland haben Pflegeheimbewohner jährlich etwa 1,7 Notaufnahmebesuche [8]. Im internationalen Vergleich werden Pflegeheimbewohner hierzulande häufiger hospitalisiert [13] und sie versterben häufiger im Krankenhaus [2]. In der Literatur werden viele dieser Notaufnahmebesuche und Krankenhausaufenthalte als vermeidbar angesehen [17, 19]. Erfolgt trotzdem ein Krankenhaustransport, sind Notaufnahmen gerade bei diesen vulnerablen Patienten die wesentlichen Weichensteller für die anschließenden Versorgungsprozesse [21]. Trotz dieser hohen Bedeutung stammen die wenigen Studien zu klinischen Charakteristika, Versorgungsprozessen und Ergebnissen von Notaufnahmebesuchen von Pflegeheimbewohnern größtenteils aus dem Ausland [4, 23].

Ziel dieser Arbeit war es deshalb, diese Lücke mit Daten einer deutschen Notaufnahme zu schließen.

## Methodik

### Setting und Datenbasis

Wir verwendeten Routinedaten des Pius-Hospitals Oldenburg, einem freigemeinnützigen Krankenhaus der Schwerpunktversorgung ohne Unfallchirurgie mit 391 vollstationären und 8 teilstationären Behandlungsplätzen. Daneben gibt es in Oldenburg 2 weitere Krankenhäuser mit ca. 1200 Betten. Oldenburg ist eine Großstadt im Nordwesten Deutschlands im Flächenland Niedersachsen mit ca. 166.500 Einwohnern, zu deren Kerngebiet insgesamt 600.000 Einwohner gehören.

Im Pius-Hospital werden Notfallpatienten im interdisziplinären Aufnahmezentrum betreut, das aus der interdisziplinären Notaufnahme mit 8 Behandlungsräumen und der interdisziplinären Aufnahmestation mit 18 Betten besteht. Dort werden alle Notfallpatienten der Kliniken für Hämatologie und internistische Onkologie, innere Medizin, Pneumologie und Gastroenterologie, Allgemein- und Viszeralchirurgie, Orthopädie und Unfallchirurgie, Strahlentherapie und Radioonkologie sowie Thorax‑, Gefäß- und endovaskuläre Chirurgie versorgt. Die digitale Dokumentation erfolgt nach dem einheitlichen Protokoll des AKTIN-Notaufnahmeregisters [3].

### Studienpopulation und analysierte Variablen

Aus dem Krankenhausinformationssystem wurden alle Pflegeheimbewohner selektiert, die im Zeitraum 06/2014 bis 05/2017 in der Notaufnahme versorgt wurden. Die Selektion erfolgte über die Adressen der Pflegeheime. Eine Altersbegrenzung gab es nicht.

Wir analysierten Informationen zum Zuweisungsweg, zur Ersteinschätzung, zu Vitalparametern sowie zum weiteren Versorgungsverlauf und zu administrativen Patientendaten. Als Informationen zum Zuweisungsweg erfassten wir die Zuweisungsart (wobei wir Hausarzt, Facharzt, Notdienst der Kassenärztlichen Vereinigung [KV] als Vertragsarzt zusammenfassten) sowie die Transportart. Die Ersteinschätzung von Symptomen und Beschwerden durch das Pflegepersonal liegt in Form des Manchester-Triage-System vor, das 5 Stufen der Dringlichkeit und damit der maximalen Arztwartezeit unterscheidet (kategorisiert in blau/grün, gelb und orange/rot). Innerhalb des Manchester-Triage-Systems werden für den Entscheidungsprozess sog. generelle Indikatoren (z. B. Lebensgefahr, Schmerzen oder Blutverlust) sowie spezielle Indikatoren für einzelne Beschwerdebilder berücksichtigt. Weiterhin haben wir die Beschwerdekategorien des Manchester-Triage-Algorithmus analysiert, um die Vorstellungsgründe abzubilden.

Bezüglich des Versorgungsverlaufs wurde erfasst ob, a) in der Notaufnahme eine Blutentnahme stattfand, b) eine Röntgendiagnostik durchgeführt wurde, c) eine stationäre Aufnahme erfolgte und d) der Bewohner während des stationären Aufenthalts verstorben ist.

Als administrative Patientendaten wurden Alter, Geschlecht, Datum und Uhrzeit des Notaufnahmebesuchs sowie die Verweildauer in der Notaufnahme und des stationären Aufenthalts erfasst.

### Statistische Analyse

Die Daten wurden deskriptiv ausgewertet. Bei dichotomen und kategorialen Variablen wurden absolute und relative Häufigkeiten bestimmt. Für stetige Variablen wurden verschiedene Lage- und Streuungsmaße (Mittelwert und Standardabweichung [SD] sowie Median und Interquartilsabstand [IQR]) berechnet.

Alle statistischen Analysen wurden mit SAS für Windows Version 9.4 (SAS Institute Inc, Cary, NC, USA) durchgeführt.

Die Studie wurde vom Datenschutzbeauftragten des Pius-Hospitals befürwortet. Es liegt ein positives Votum der Medizinischen Ethikkommission der Carl von Ossietzky Universität vor (Nr. 2016-128).

## Ergebnisse

### Baselinecharakteristika

Im Untersuchungszeitraum wurden insgesamt 47.270 Patienten in der Notaufnahme behandelt (mittleres Alter: 58,8 Jahre; 53,5 % weiblich). Der überwiegende Anteil wurde in die leichten Triagekategorien blau/grün (52 %) und lediglich 12 % als orange/rot eingestuft. Die Aufnahmequote lag bei 47,7 % und die durchschnittliche Verweildauer bei 7,1 Tagen.

Von diesen 47.270 Notaufnahmepatienten waren 1676 Pflegeheimbewohner (3,6 %). Pflegeheimbewohner waren durchschnittlich 78,8 Jahre alt und zu 62,9 % weiblich (Tab. 1). Die Bewohner wurden in etwa gleich häufig in die leichten Triagekategorien blau/grün (39,7 %) sowie dringend als gelb eingestuft (38,3 %). Das mit Abstand führende Beschwerdebild war Atemnot bei Erwachsenen (31,5 %). Die Vitalparameter, die am häufigsten Auffälligkeiten zeigten, waren eine erhöhte Herz- sowie Atemfrequenz. Die durchschnittliche Verweildauer in der Notaufnahme lag bei ungefähr 3 h. Insgesamt wurden 81,0 % der Pflegeheimbewohner internistisch versorgt.

**Table 1:** 

	Ambulant	Stationär	Gesamt
	(*n* = 264; 15,8 %)	(*n* = 1412; 84,2 %)	(*n* = 1676; 100 %)
*Weibliches Geschlecht (n* *=* *1676)*	65,2 %	62,5 %	62,9 %
*Alter in Jahren (n* *=* *1676)*			
Mittelwert (SD)	73,4 (19,3)	79,8 (13,6)	78,8 (14,8)
Median [IQR]	80 [65–87]	83 [76–89]	83 [74–89]
*Triagekategorie* ^a^* (n* *=* *1676)*			
Blau/grün	67,8 %	34,5 %	39,7 %
Gelb	24,2 %	40,9 %	38,3 %
Orange/rot	8,0 %	24,6 %	22,0 %
*Beschwerdekategorie (n* *=* *1676)*			
Atemnot bei Erwachsenen	10,6 %	35,4 %	31,5 %
Abdominelle Schmerzen bei Erwachsenen	9,5 %	15,9 %	14,9 %
Extremitätenprobleme	9,2 %	41,3 %	14,3 %
Generelle Indikatoren	10,2 %	7,2 %	7,6 %
Unwohlsein bei Erwachsenen	6,4 %	7,9 %	7,6 %
Gastrointestinale Blutung	1,1 %	6,6 %	5,7 %
Andere	20,8 %	17,9 %	19,4 %
*Vitalparameter*			
Systolischer Blutdruck ≤ 100 mm Hg (*n* = 1577)	3,1 %	11,2 %	9,9 %
Systolischer Blutdruck ≥ 180 mm Hg (*n* = 1577)	7,8 %	8,8 %	8,6 %
Herzfrequenz ≤ 60/min (*n* = 1618)	10,9 %	7,9 %	8,3 %
Herzfrequenz ≥ 100/min (*n* = 1618)	13,2 %	23,2 %	21,6 %
Atemfrequenz ≥ 21/min (*n* = 1403)	18,0 %	38,2 %	35,5 %
Sauerstoffsättigung ≤ 90 % (*n* = 1616)	3,2 %	12,3 %	10,9 %
Temperatur ≥ 38,5° (*n* = 1588)	0,8 %	4,7 %	4,0 %
*Wochentag des Notaufnahmebesuchs (n* *=* *1675)*			
Montag bis Freitag	83,3 %	79,2 %	79,9 %
Samstag bis Sonntag	16,7 %	20,8 %	20,1 %
*Zuweisungsart (n* *=* *1139)*			
Vertragsarzt	46,9 %	39,1 %	40,3 %
Notarzt	3,9 %	12,4 %	11,1 %
Ohne	43,6 %	42,7 %	42,8 %
Andere	5,6 %	5,8 %	5,8 %
*Verweildauer in der Notaufnahme, in min (n* *=* *1676)*			
Mittelwert (SD)	188,3 (89,0)	171,6 (97,0)	174,3 (96,0)
Median [IQR]	180 [120–240]	156 [107,5–214]	162 [112–222]

^a^ *Blau* nicht dringend (Wartezeit max. 120 min), *grün* normal (max. 90 min), *gelb* dringend (max. 30 min), *orange* sehr dringend (max. 10 min), *rot* sofort (keine Wartezeit)

Ausschließlich ambulant versorgte Bewohner waren durchschnittlich jünger und weniger dringlich triagiert. Auffällig ist vor allem, dass sie deutlich häufiger Extremitätenprobleme als Beschwerdebild aufwiesen (dabei handelte es sich vor allem um den Ausschluss einer Thrombose).

### Zuweisung, Transport und Zeitpunkt der Notaufnahmekontakte

**Figure 1:**
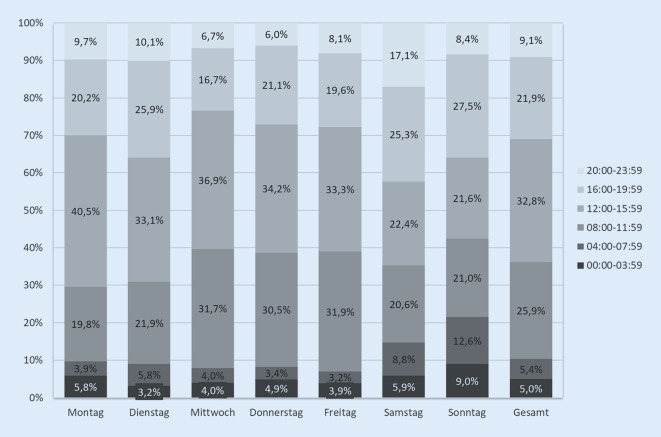

Die Notaufnahmebesuche verteilten sich zu je 15–17 % auf die einzelnen Wochentage und zu jeweils 10 % auf Samstage und Sonntage. Betrachtet man die Uhrzeiten des Eintreffens, erfolgten 80,6 % der Besuche zwischen 8.00 und 20.00 Uhr. Dieser Anteil ist mit 68,2 % bzw. 70,1 % an den Wochenenden etwas niedriger (Abb. 1). Insgesamt 57,6 % der Besuche konzentrierten sich auf die Zeit zwischen 10.00 und 17.00 Uhr.

**Figure 2:**
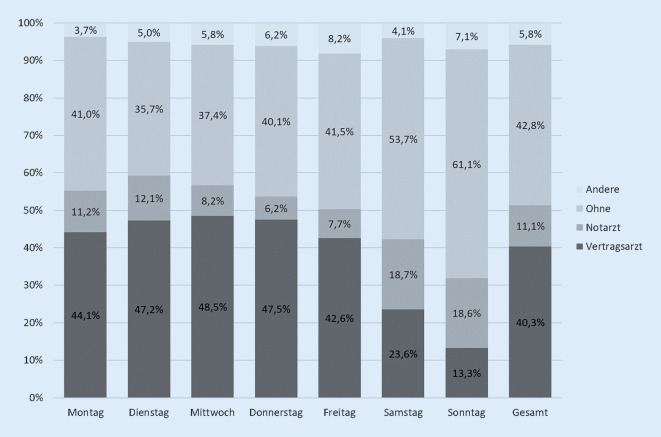

Für 1139 der 1676 Besuche lagen Informationen zur Zuweisungsart vor (68,0 %). Insgesamt wurden 40,3 % durch Vertragsärzte eingewiesen, dieser Anteil lag in der Woche zwischen 43 und 48 % und am Wochenende niedriger (Abb. 2).

Für 1032 Besuche lagen Informationen zur Art des Transports vor (61,6 %). Insgesamt 75,7 % kamen mit dem Krankentransport- bzw. Rettungswagen (zwischen 72 und 79 % nach Wochentagen), 10,4 % mit Begleitung des Notarztes (zwischen 6 und 18 % mit höheren Werten am Wochenende) und der verbleibende Teil wurde anderweitig transportiert (14,0 %, zwischen 8 und 18 % mit niedrigeren Werten am Wochenende).

### Versorgungsverlauf und Verweildauer

Für 94,2 % bzw. 46,2 % wurden in der Notaufnahme eine Blutentnahme bzw. eine radiologische Diagnostik durchgeführt.

Insgesamt wurden 84,2 % der Pflegeheimbewohner stationär aufgenommen, die durchschnittliche Verweildauer lag bei 7,4 Tagen (SD: 7,7). Die Verweildauer betrug genau einen Tag bei 21,1 %, 2–3 Tage bei 14,6 % sowie 4 und mehr Tage bei 64,4 % (s. eTabelle 1 für den Vergleich dieser Gruppen). Mit längerer Verweildauer wurde ein größerer Anteil durch Vertragsärzte eingewiesen, gleichzeitig ging der Anteil ohne Einweisung von 51,9 % bzw. 49,3 % (ein bzw. 2–3 Tage) auf 37,4 % (≥4 Tage) zurück. Keine eindeutigen Trends zeigten sich z. B. bei der Triagierung, dem Alter, den Wochentagen und Uhrzeiten. Bewohner mit kürzerer Liegedauer zeigten eine breitere Verteilung der Beschwerdebilder.

### Versterben im Krankenhaus

Von allen stationär aufgenommenen Bewohnern verstarben 10,3 % während des Krankenhausaufenthalts. Von diesen verstarben 28,2 % noch am Aufnahmetag und 47,9 % binnen der ersten 3 Tage des Aufenthalts.

Verstorbene unterschieden sich in wesentlichen Charakteristika von den aus dem Krankenhaus entlassenen Bewohnern (eTabelle 2). Sie waren durchschnittlich älter, die Notaufnahmebesuche waren häufiger am Wochenende und eine Einweisung durch einen Vertragsarzt erfolgte seltener. Im Krankenhaus verstorbene Bewohner waren häufiger als dringlich behandlungsbedürftig triagiert, wiesen häufiger Atemnot als führendes Beschwerdebild sowie eine niedrige Sauerstoffsättigung bzw. eine hohe Atem- und Herzfrequenz auf.

## Diskussion

Insgesamt 3,6 % aller Notaufnahmebesuche entfielen auf Pflegeheimbewohner, von diesen wurden fast 85 % stationär aufgenommen. Lediglich 40 % wurden durch einen Vertragsarzt eingewiesen. Jeder 10. stationär aufgenommene Bewohner verstarb während des Krankenhausaufenthalts, etwa ein Drittel davon noch am Aufnahmetag. Verstorbene Bewohner wurden häufiger am Wochenende vorgestellt, seltener durch einen Vertragsarzt eingewiesen und waren häufiger als dringlich behandlungsbedürftig triagiert.

### Vergleich der Ergebnisse mit der Literatur

Mit 3,6 % machen Pflegeheimbewohner einen nicht unerheblichen Anteil aller Notaufnahmebesuche aus, der höher liegt als in einer amerikanischen Studie [4]. Insgesamt erfolgten nur etwa 40 % der Transporte mit Überweisung eines Vertragsarztes, obwohl diese oft während der Praxisöffnungszeiten stattfanden. Dies untermauert Ergebnisse einer anderen Studie, dass die Entscheidung zum Krankenhaustransport oftmals allein vom Pflegepersonal getroffen wird [18]. Nach einer Befragung sind auch 54 % der Hausärzte der Meinung, dass das Pflegepersonal solche Entscheidungen zu häufig ohne ärztliche Rücksprache trifft [9]. Interessanterweise waren in dieser Studie nur 9 % der Heimleitungen der gleichen Ansicht, die jedoch deutlich häufiger eine bessere Erreichbarkeit des Hausarztes forderten. Dass durch eine bessere Kommunikation zwischen Pflegepersonal und Hausärzten Krankenhaustransporte vermieden werden können, befürwortet jedoch ein großer Teil beider Berufsgruppen (Hausärzte: 91 %; Heimleitungen: 64 %; [9]). Auch ein systematischer Review hebt in diesem Zusammenhang die Notwendigkeit einer besseren Kooperation und Kommunikation zwischen qualifiziertem Pflegepersonal und Hausärzten sowie der Verfügbarkeit von Hausärzten als wesentliche Faktoren hervor [17]. Eine hohe Barriere, um diese Maßnahmen umzusetzen, ist in Deutschland sicherlich die Tatsache, dass durchschnittlich 8,6 verschiedene Hausärzte an der medizinischen Versorgung der Bewohner jeweils eines Pflegeheims beteiligt sind [20].

Weiterhin zeigte sich, dass Pflegeheimbewohner in 10,3 % der Krankenhausaufenthalte verstarben. Dieser Anteil ist nahezu identisch zu einer Studie mit Daten der AOK Bremen/Bremerhaven (9,6 %; [8]). Insgesamt versterben in Deutschland etwa ein Drittel aller Pflegeheimbewohner im Krankenhaus und etwa die Hälfte wird innerhalb des letzten Lebensmonats stationär behandelt [15]. Diese Anteile haben sich während der letzten 10 Jahre kaum verändert [16], jedoch stieg zeitgleich die Bedeutung des Settings Pflegeheim für die Versorgung am Lebensende [12]. Ein systematischer Review (*n* = 35 Studien) fand, dass Pflegeheimbewohner in Deutschland im internationalen Vergleich häufiger in der letzten Lebensphase im Krankenhaus behandelt wurden [2]. In der Literatur werden insbesondere kurze Krankenhausaufenthalte am Lebensende von Pflegeheimbewohnern als kritisch und oft vermeidbar angesehen [10, 17]. In diesem Zusammenhang ist der Anteil von 28,2 % aller Verstorbenen, deren stationäre Aufenthaltsdauer genau einen Tag betrug, in dieser Studie bemerkenswert. Andere Studien aus Deutschland finden Werte zwischen 10 und 11 % [14, 16]. Allerdings zeigen auch diese Studien, dass die stationäre Aufenthaltsdauer vor Tod bei etwa einem Drittel maximal 3 Tage betrug ([14, 16], hier 48 %) und untermauern damit dieses Versorgungsproblem. Mit 71 % schätzt auch die große Mehrheit der Hausärzte ein, dass Pflegeheimbewohner am Ende des Lebens zu häufig im Krankenhaus behandelt werden [1]. Bewohner, die während des Aufenthalts versterben, werden nach unserer Studie jedoch in lediglich 31 % der Fälle überhaupt durch einen Vertragsarzt eingewiesen. Hier zeigt sich nochmals die Notwendigkeit von besserer interdisziplinärer Kommunikation, aber auch von Advance Care Planning (ACP) idealerweise unter Einbezug der Angehörigen bzw. die Notwendigkeit aussagekräftiger Patientenverfügungen [17]. Zwar wurden 2018 in § 132g SGB V Möglichkeiten zur gesundheitlichen Versorgungsplanung für die letzte Lebensphase geschaffen, wodurch Heime zusätzliche Leistungen von ACP erbringen und abrechnen können, jedoch läuft die Umsetzung schleppend. Insgesamt besteht für geriatrische Patienten und Pflegeheimbewohner, bei denen nichtonkologische Krankheitsbilder führend sind, in Deutschland eine Unterversorgung im Bereich palliativer Angebote [22] sowie eine erhebliche regionale Heterogenität in deren Verfügbarkeit bzw. Nutzung [6].

Auffällig ist auch, dass Pflegeheimbewohner im Vergleich zu allen Notaufnahmepatienten deutlich dringlicher triagiert wurden, was jedoch zur internationalen Literatur passt [5, 7]. Eine mögliche Erklärung ist, dass die Multimorbidität dieser Klientel eher dazu führt, einen rascheren Arztkontakt als notwendig zu erachten. Dies zeigt sich insbesondere auch für im Krankenhaus verstorbene Bewohner, von denen nahezu die Hälfte als orange bzw. rot triagiert wurden und Atemnot das führende Beschwerdebild war. Dies lässt vermuten, dass klassische Instrumente zur Ersteinschätzung gerade bei diesen Patienten zu einer Über- bzw. Fehltriagierung führen, da sie auf akut lebensbedrohliche Symptome wie Atemnot oder Vigilanzveränderungen fokussieren, die in einer palliativen Situation jedoch einen gänzlich anderen Therapieauftrag auslösen. Entsprechend sollten für solche Patienten zusätzliche Instrumente zur Einschätzung eines geriatrischen bzw. palliativen Versorgungsbedarfs etabliert werden. Ohne den Einsatz umfassender Assessments besteht bei geriatrischen Patienten in der Notaufnahme die Gefahr, wesentliche versorgungsrelevante Informationen zu übersehen.

### Stärken und Schwächen

Wesentliche Stärke dieser Arbeit ist die große Stichprobe von fast 1700 Notaufnahmebesuchen von Pflegeheimbewohnern, für die nach einem einheitlichen Notaufnahmeprotokoll generierte elektronische Daten in vergleichsweise großer Informationstiefe vorliegen (z. B. auch klinische Variablen zu Triagekategorien, Beschwerdegruppen sowie Vitalparametern). Andererseits fehlen wesentliche Informationen, z. B. zu körperlichen und kognitiven Einschränkungen. Diagnosen liegen ausschließlich für stationäre Aufnahmen vor, jedoch sind für alle Patienten Beschwerdekategorien vorhanden. Obwohl die Erfassungsqualität insgesamt sehr gut ist, fehlen bei einzelnen Variablen Angaben. So liegen bei der Einweisungsart für 32 % keine Angaben vor. Dies betrifft jedoch ausschließlich die früheren Jahre, in 2017 gab es dabei keine fehlenden Werte mehr. Wertet man ausschließlich das Jahr 2017 aus (*n* = 224), finden sich identische Verteilungen (41,5 % ohne sowie 41,5 % durch Vertragsärzte) wie für alle Jahre (42,8 % ohne sowie 40,3 % durch Vertragsärzte). Dies trifft auch auf die Art des Transports zu. Somit ist nicht davon auszugehen, dass durch die fehlenden Werte systematische Verzerrungen entstanden sind.

Wesentliche Schwäche der Studie ist, dass die Erhebung in lediglich einem Krankenhaus durchgeführt wurde, in dem die Fachrichtungen Unfallchirurgie und Neurologie nicht vorhanden sind. Dies bedeutet, dass in der Studie fast keine unfallchirurgischen Krankheitsbilder erfasst sind, die in anderen Studien einen relevanten Anteil der vorgestellten Pflegeheimbewohner ausmachen [4, 23]. Weiterhin stellt sich auch aufgrund der städtischen Lage die Frage der Generalisierbarkeit der Ergebnisse. Um diese zu erreichen, wäre der Aufbau eines deutschlandweiten Notaufnahmeregisters notwendig, das mit dem AKTIN-Projekt bereits begonnen wurde [3] und wodurch für diese Studie umfangreiche, elektronische und strukturiert erfasste Daten vorlagen. Allerdings wäre die durchgeführte Analyse aufgrund der fehlenden Zuordnung des Settings Pflegeheim nicht innerhalb der Struktur des AKTIN-Projekts möglich. Diese Zuordnung erfolgte hier über eine zusätzliche Adressselektion aus dem Krankenhausinformationssystem. Es wäre allerdings wünschenswert, wenn auch das AKTIN-Notaufnahmeregister um ein zusätzliches Feld zum Setting Pflegeheim ergänzt würde. Eine weitere mögliche Limitation unserer Selektion könnte sein, dass die Adressen von Pflegeheimbewohnern noch nicht umgemeldet gewesen sein könnten. Somit würde der Anteil an Pflegeheimbewohnern unterschätzt. Einzelne stratifizierte Auswertungen wurden mit teils niedrigen Fallzahlen durchgeführt, wodurch die Ergebnisse weniger robust sind.

## Fazit für die Praxis

Die Studie zeigt die hohe Bedeutung des Settings Notaufnahme für die Versorgung von Pflegeheimbewohnern. Sie zeigt aber auch die Probleme auf verschiedenen Seiten im Umgang mit dieser Patientengruppe. Einerseits werden Vertragsärzte nur selten in die Transportentscheidung eingebunden, insbesondere am Lebensende, obwohl der Großteil der Transporte während der Praxisöffnungszeiten erfolgt. Hier sollten zukünftig bessere Lösungen der interdisziplinären Kooperation insbesondere mit Hausärzten sowie Stärkungen der Palliativstrukturen im Heim gefunden werden. Gleichzeitig wird auch durch die Notaufnahmen ein sehr hoher Anteil der Bewohner stationär aufgenommen. Hier scheint neben der Triagierung ein weiteres Assessment in Bezug auf den geriatrischen und palliativen Versorgungsbedarf sowie hinterlegte Prozesse erforderlich, die auch eine weitere ambulante Versorgung ermöglichen, so denn die ambulanten Strukturen die weitere palliative Versorgung gewährleisten können.

### Supplementary Information





