# Supplementary material for: Emergency department visits by nursing home residents: analysis of routine data from an urban hospital
Source: Med Klin Intensivmed Notfmed. 2022 Sep 7;118(7):549–55. [Article in German] doi: 10.1007/s00063-022-00952-2 (PMC10564807; doi:10.1007/s00063-022-00952-2)
Supplement: Supplementary file 1 [file 63_2022_952_MOESM1_ESM.docx]

|  | 1 Tag  (n=290; 21,1%) | 2-3 Tage  (n=200; 14,6%) | 4+ Tage  (n=885; 64,4%) |
| --- | --- | --- | --- |
| Weibliches Geschlecht (n=1.375) | 62,8% | 65,0% | 62,3% |
| Alter in Jahren (n=1.375) |  |  |  |
| Mittelwert (SD) | 78,5 (15,9) | 79,2 (15,3) | 80,5 (12,1) |
| Median [IQR] | 84 [74-89] | 83 [74-90] | 84 [76-89] |
| Triage-Kategorie^a)^ (n=1.375) |  |  |  |
| Blau/ grün | 38,3% | 44,0% | 31,2% |
| Gelb | 37,2% | 30,0% | 44,9% |
| Orange/ rot | 24,5% | 26,0% | 24,0% |
| Beschwerdekategorie (n=1.375) |  |  |  |
| Atemnot bei Erwachsenen | 23,8% | 25,0% | 41,5% |
| Abdominelle Schmerzen bei Erwachsenen | 16,9% | 20,0% | 14,7% |
| Extremitätenprobleme | 8,3% | 9,5% | 9,3% |
| Unwohlsein bei Erwachsenen | 7,6% | 7,5% | 8,1% |
| Generelle Indikatoren | 9,3% | 7,0% | 6,4% |
| Gastrointestinale Blutung | 7,9% | 10,0% | 5,4% |
| Andere | 26,2% | 21,0% | 14,6% |
| Vitalparameter |  |  |  |
| Systolischer Blutdruck ≤ 100mmHg (n=1.284) | 11,4% | 10,0% | 11,3% |
| Systolischer Blutdruck ≥ 180mmHg (n=1.284) | 10,2% | 12,2% | 7,6% |
| Herzfrequenz ≤ 60/min (n=1.325) | 9,0% | 5,7% | 7,5% |
| Herzfrequenz ≥ 100/min (n=1.325) | 17,6% | 21,4% | 25,6% |
| Atemfrequenz ≥ 21/min (n=1.182) | 33,5% | 32,7% | 40,8% |
| Sauerstoffsättigung ≤ 90% (n=1.326) | 9,8% | 8,8% | 13,8% |
| Temperatur ≥ 38,5° (n=1.300) | 1,5% | 4,8% | 5,5% |
| Wochentag des Notaufnahmebesuchs (n=1.374) |  |  |  |
| Montag-Freitag | 78,6% | 71,0% | 81,2% |
| Samstag-Sonntag | 21,4% | 29,0% | 18,9% |
| Uhrzeit des Notaufnahmebesuchs (n=1.374) |  |  |  |
| 00:00-03:59 Uhr | 7,8% | 5,0% | 5,0% |
| 04:00-07:59 Uhr | 5,5% | 7,5% | 5,8% |
| 08:00-11:59 Uhr | 25,9% | 19,0% | 26,4% |
| 12:00-15:59 Uhr | 33,8% | 32,0% | 31,2% |
| 16:00-19:59 Uhr | 20,0% | 22,5% | 22,6% |
| 20:00-23:59 Uhr | 7,6% | 14,0% | 9,1% |
| Zuweisungsart (n=931) |  |  |  |
| Vertragsarzt | 29,1% | 35,5% | 43,4% |
| Notarzt | 12,0% | 10,1% | 13,5% |
| Ohne | 51,9% | 49,3% | 37,4% |
| Andere | 6,3% | 5,1% | 5,6% |
| Im Krankenhaus verstorben (n=1.375) | 13,8% | 14,0% | 8,4% |

^a)^ blau=nicht dringend (Wartezeit max. 120 Min.), grün=normal (max. 90 Min.), gelb=dringend (max. 30 Min.), orange=sehr dringend (max. 10 Min.), rot=sofort (keine Wartezeit)

eTabelle 1: Vergleich der Charakteristika von stationär aufgenommenen Pflegeheimbewohnern nach Verweildauer (n=1.375; bei n=37 Patienten fehlen Angaben zur Verweildauer)
