# Supplementary material for: Emergency department visits by nursing home residents: analysis of routine data from an urban hospital
Source: Med Klin Intensivmed Notfmed. 2022 Sep 7;118(7):549–55. [Article in German] doi: 10.1007/s00063-022-00952-2 (PMC10564807; doi:10.1007/s00063-022-00952-2)
Supplement: Supplementary file 2 [file 63_2022_952_MOESM2_ESM.docx]

|  | Verstorben  (n=142; 10,3%) | Nicht verstorben  (n=1.233; 89,7%) |
| --- | --- | --- |
| Weibliches Geschlecht (n=1.375) | 69,0% | 62,0% |
| Alter in Jahren (n=1.375) |  |  |
| Mittelwert (SD) | 84,2 (10,7) | 79,4 (13,7) |
| Median [IQR] | 87 [80-92] | 83 [75-89] |
| Triage-Kategorie^a)^ (n=1.375) |  |  |
| Blau/ grün | 19,7% | 36,3% |
| Gelb | 32,4% | 42,1% |
| Orange/ rot | 47,9% | 21,7% |
| Beschwerdekategorie (n=1.375) |  |  |
| Atemnot bei Erwachsenen | 47,9% | 33,9% |
| Abdominelle Schmerzen bei Erwachsenen | 11,3% | 16,5% |
| Extremitätenprobleme | 10,6% | 8,9% |
| Unwohlsein bei Erwachsenen | 7,8% | 8,0% |
| Generelle Indikatoren | 7,0% | 7,1% |
| Gastrointestinale Blutung | 4,9% | 6,8% |
| Andere | 15,5% | 18,9% |
| Vitalparameter |  |  |
| Systolischer Blutdruck ≤ 100mmHg (n=1.284) | 24,6% | 9,6% |
| Systolischer Blutdruck ≥ 180mmHg (n=1.284) | 1,5% | 9,7% |
| Herzfrequenz ≤ 60/min (n=1.325) | 5,9% | 7,4% |
| Herzfrequenz ≥ 100/min (n=1.325) | 47,8% | 20,5% |
| Atemfrequenz ≥ 21/min (n=1.182) | 60,3% | 35,6% |
| Sauerstoffsättigung ≤ 90% (n=1.326) | 26,9% | 10,6% |
| Temperatur ≥ 38,5° (n=1.300) | 7,3% | 4,2% |
| Wochentag des Notaufnahmebesuchs (n=1.374) |  |  |
| Montag-Freitag | 69,0% | 80,4% |
| Samstag-Sonntag | 31,0% | 19,6% |
| Zuweisungsart (n=931) |  |  |
| Vertragsarzt | 31,0% | 40,1% |
| Notarzt | 18,0% | 12,0% |
| Ohne | 42,0% | 42,5% |
| Andere | 9,0% | 5,3% |
| Verweildauer in Notaufnahme, in Min. (n=1.375) |  |  |
| Mittelwert (SD) | 162,2 (120,1) | 170,9 (90,1) |
| Median [IQR] | 131 [90-202] | 158 [111-214] |
| Verweildauer in Krankenhaus (n=1.375) |  |  |
| 1 Tag | 28,2% | 20,3% |
| 2-3 Tage | 19,7% | 14,0% |
| 4+ Tage | 52,1% | 65,8% |

^a)^ blau=nicht dringend (Wartezeit max. 120 Min.), grün=normal (max. 90 Min.), gelb=dringend (max. 30 Min.), orange=sehr dringend (max. 10 Min.), rot=sofort (keine Wartezeit)

eTabelle 2: Vergleich von im Krankenhaus verstorbenen und nicht verstorbenen Pflegeheimbewohnern (n=1.375; bei n=37 Patienten fehlen Angaben zur Entlassart)
